# Heavy Metals and Human Health: Possible Exposure Pathways and the Competition for Protein Binding Sites

**DOI:** 10.3390/molecules26196060

**Published:** 2021-10-07

**Authors:** Danuta Witkowska, Joanna Słowik, Karolina Chilicka

**Affiliations:** Institute of Health Sciences, University of Opole, 68 Katowicka St., 45-060 Opole, Poland; joanna.slowik@uni.opole.pl (J.S.); karolina.chilicka@uni.opole.pl (K.C.)

**Keywords:** heavy metals, proteins, exposure, interactions, bioremediation

## Abstract

Heavy metals enter the human body through the gastrointestinal tract, skin, or via inhalation. Toxic metals have proven to be a major threat to human health, mostly because of their ability to cause membrane and DNA damage, and to perturb protein function and enzyme activity. These metals disturb native proteins’ functions by binding to free thiols or other functional groups, catalyzing the oxidation of amino acid side chains, perturbing protein folding, and/or displacing essential metal ions in enzymes. The review shows the physiological and biochemical effects of selected toxic metals interactions with proteins and enzymes. As environmental contamination by heavy metals is one of the most significant global problems, some detoxification strategies are also mentioned.

## 1. Introduction

Some metal ions, such as Na(I), K(I), Mg(II), and Ca(II), are essential nutrients required for body function, others can be toxic even at very low concentrations. Regarding metals, micronutrients such as zinc, copper, iron, and manganese can also cause adverse effects when present in excessive levels, or if their homeostasis is disturbed by some factors [1,2]. However, normally these ions play important and beneficial roles in human metabolism.

Heavy metal ions can act as either cofactors or inhibitors in enzymatic pathways. It has been estimated that about half of all enzymes require a metal cofactor to be active and functional [3,4].

There are few definitions of “heavy metals”. Generally, this term refers to metals and metalloids with relatively high densities (more than 5 g/cm^3^), bioaccumulative potential along the food chain, and usually high toxicity to living organisms. Some researchers suggest replacing the controversial term “heavy metals” with “potentially toxic elements” [5].

This group comprises toxic metals, including cadmium (Cd), lead (Pb), nickel (Ni), chromium (Cr), mercury (Hg), and metalloids, such as arsenic (As), from both natural sources and industry.

It is well known that exposure to xenobiotic metals can cause gastrointestinal, respiratory, cardiovascular, reproductive, renal, hemopoietic, and neurological disorders [6,7].

Some heavy metals stimulate through different pathogenetic links the progression of cancers and reduce their sensitivity to treatment [8,9]. Oxidative stress (rising level of oxidative damage in a cell) caused by these metals destroys lipids, proteins and DNA molecules, and supports carcinogenesis. A wider discussion on this topic can be found in recent reviews [10,11,12].

**Figure 1:**
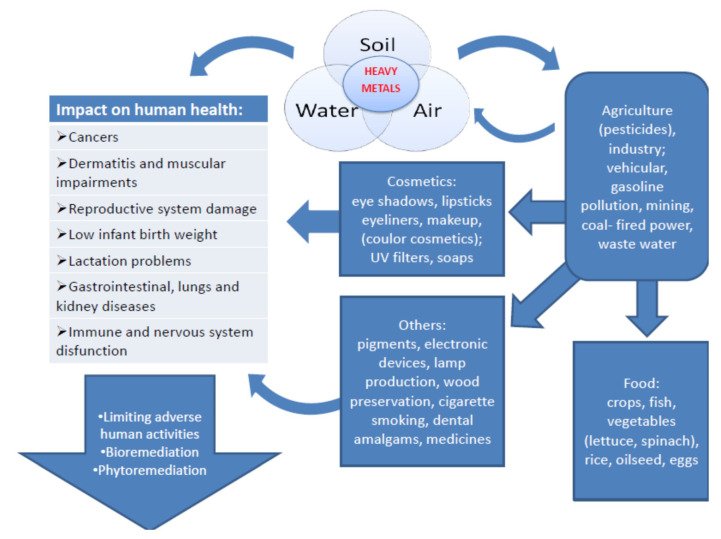
Routes of exposure, the impact of toxic metals on human health, and the ways of limiting the risk caused by contact with these elements (large arrow on the left). These adverse effects are caused by direct exposure to the toxic metals in the environment or indirectly due to anthropogenic activity.

The possible routes of exposure and the impact of heavy metals on human health are shown in Figure 1. A detailed description of their mechanism of action is provided below.

It is worth mentioning, that simultaneous exposure to a mixture of xenobiotics (heavy metals, pesticides, and other toxins) may have a cumulative effect [13,14].

The permissible levels of different heavy metal ions, set by World Health Organization (WHO) and European Medical Agency (EMA), range from ppt to ppm. As of 1 June 2020, arsenic (As), cadmium (Cd), lead (Pb) and mercury (Hg) are within the 10 chemicals of major public health concern, as shown recently in the WHO website [15]. Despite the fact that the toxicity of these elements is well-known, their various technological, medical and agricultural, applications cause still a huge threat to human health.

Despite many efforts of agencies such as the EMA, WHO or European Environment Agency (EEA), industrial development leads to rising levels of toxic metals in soil, water and air, constituting a direct and/or indirect threat for human health. This review provides a synthesis of existing knowledge on the sources of heavy metals toxic for humans, such as arsenic (As), cadmium (Cd), lead (Pb), mercury (Hg), and nickel (Ni), and their impact on human health, with a special emphasis on toxic metals’ interactions with proteins. Biological detoxification strategies are also briefly described.

## 2. Heavy Metals in Food and Water

There are many ways heavy metals can enter the environment and agriculture. Heavy metal contamination of foods originates by weathering of the bedrock, air pollution directly, as well as soil irrigation with polluted waters and polluting groundwater [16]. Environmental contamination, primarily by industrial and human activity, refers to soil or groundwater that are the most common access routes of heavy metals such as lead, mercury, chromium, arsenic and cadmium. Reports have shown that increased amounts of heavy metals in the human daily diet are related to region, available food products and industry. Occasionally, a metal is not released into the environment, but human industrial activities result in exposure to those which are naturally occurring. For example, in the vicinity of a mine, soil contamination can be observed, which can result in the presence of heavy metals in local crops [17]. Certain regions and related industries are known to be specific for the emergence of these heavy metals in high concentrations, i.e., in China, areas surrounding coal-fired power plants are polluted by mercury up to 10 times more than the average soil sample 55 km away from these places [18].

Mercury has a significant influence on the human body. Reports have shown that mercury is largely ingested through the consumption of fish: marine, freshwater and starfish [18,19,20]. A study conducted in China revealed that mercury levels in children’s blood varies depending on the region and on the rate of fish consumption [21]. The level of organic mercury (e.g., methylmercury) depends on the species of fish [22]. Bioaccessibility of mercury in food differs between raw and cooked foods and can be reduced by drinking green or black tea and black coffee during a meal [23,24], which can be correlated to the presence of phytates and tannins. The presence of mercury has also been confirmed in lettuce, water spinach, amaranth, rice, etc. [25]. Li et al. examined the impact of coal-fired power plants on vegetables and crops, and showed that mercury pollution is more prominent in leaves than roots [18].

However, absorption of mercury in the gastrointestinal track is approximately 7% to 15%, compared with 80% when inhaled [26]. This comparison shows that inhaling mercury vapors, which occurs, e.g., during small-scale gold mining, silver-ore work and dental work with amalgams, is dangerous. Mercury causes neurological problems and impairs kidney function. Additionally, a correlation has been determined between infertility and higher blood mercury levels [27].

Lead is another heavy metal which has an important impact on human health. Human poisoning is often associated with lead gasoline, mining and battery recycling, but the source of the poisoning may be unexpected in terms of wall paints or binders in sewer pipes [28,29]. In the Middle East, especially Egyptian villages, inhabitants produce household flour (the main component of their diet) using a stone mill, however, the stone is held with a lead binder, which causes contamination [30]. Lead and cadmium cause the loss of calcium and abnormal bone metabolism, leading to osteomalacia. Exposure to lead is especially dangerous for children under six years of age, as it disturbs development, growth and differentiation of nerve cells, and it causes damage of bone tissue. In the 1980s, the National Health and Nutrition Examination Survey (NHANES) indicated that an estimated 88% of children aged 1–5 years had blood lead levels (BLLs) ≥ 10 µg/dL. Since that time, the numbers have been rapidly decreasing. Recently, the new reference value of 3.5 µg/dL has been recommended to the American Center for Disease Control and Prevention (CDC), based on the 98th percentile of the 2011–2014 NHANES data [31]. Blood lead screening programs play an important role in reducing exposure risks, especially at the population level [32].

Cadmium has been found to greatly increase the risk of cardiovascular disease by smoking. Li et al. showed the effects of cadmium in tobacco as a cardiovascular disease factor [33]. Leafy vegetables, oilseeds, crops, organ meat, and nuts are known to contain high levels of cadmium [34]. In China, cadmium is mainly ingested via consumption of rice. Furthermore, the dismantling of Chinese e-waste exposes those in the vicinity to higher concentrations of cadmium, and those that consume rice grown in the area display a 60% increase in hazard quotient [35].

Food contamination with cadmium can come from natural sources, as well as from sewage and fertilization that gets through groundwater and runoff from fields into water bodies, causing the presence of cadmium in fish and oysters. One third of the total Cd(II) amount in the human body is accumulated in the liver and in the kidneys.

Due to the harmfulness of cadmium, which promotes serious health issues even after short-term exposure, complete avoidance is recommended. The adverse effects of chronic exposure to cadmium ions are as follows: anemia, insomnia, kidney damage, bladder and prostate cancers, and osteoporosis, among others [36].

However, unexpected ingestion of cadmium may originate from sources such as pigments and food dyes [37]. Additionally, studies have shown that a vegetarian diet possesses higher levels of cadmium compared to a nonvegetarian diet [33]. Spungen revealed that the highest ratio of cadmium is in vegetables and plant products such as sunflower seeds, spinach and potatoes, which can exceed 100 µg/kg of cadmium [38].

Consumption of cadmium has a particularly negative effect on fertility, moreover, it can cause osteotoxicity and cardiovascular diseases [39]. Furthermore, it has been shown that iron deficiencies are associated with increased absorption of divalent metals, including cadmium, cobalt and manganese [40,41]. This condition was also confirmed in breastfed infants, whose iron-poor diets were associated with increased levels of lead, cobalt and cadmium [42].

Chromium occurs as trivalent chromium and hexavalent chromium in the environment. In food, chromium is present in its low-toxicity trivalent form. However, in changing conditions (e.g., pH) chromium(III) can convert to chromium(VI). Trivalent chromium is found in nuts, broccoli, whole-grain products, and eggs [43]. Studies have examined the impact of chromium on glucose metabolism and fat loss. Hexavalent chromium is toxic and carcinogenic. Chromium(VI) is primarily generated by human activity, and is used in leather tanning, chromium plates, wood preservation, etc. Areas with tanning factories are frequently contaminated by chromium(VI) where sewage waste affects the composition of groundwater and consequently soil [44]. In Kanpur, India, drinking water contains high concentrations of chromium(VI) exceeding the average by approximately 390-fold and causes digestive, skin and ocular problems [45].

Arsenic is a well-known toxin and carcinogen. It can be found in the air, water and soil. Hopenhayn et al. examined the association between consumption of arsenic in drinking water and infant birth weight. Higher dosage of arsenic negatively affected children’s weight by approximately 57 g [46]. Geothermal activity in some regions promotes increased levels of arsenic.

In some countries, the CCA (copper-chromium-arsenic) preservative substance has been used to treat timber. This leads to the presence of heavy-metal pollution in the soil. Hence, CCA is the source and cause of heavy metals, such as arsenic, in food [47]. Singh and Ghosh tested arsenic levels in water, soil and food, where the concentration of arsenic was much higher than the WHO’s recommended limit for drinking-water (10 μg/L). Moreover, residents of contaminated areas are constantly exposed to cumulative effects of consumed arsenic from wheat, water and animal products [48].

The most common way of poisoning and intake of heavy metals is the daily consumption of contaminated food. Studies have proven that pollution of the main dietary ingredients is the primary cause of adverse effects. Orisakwe et al. showed that residents of South-East Nigeria who consumed vegetables and rice contaminated with high ratio of heavy metals, exceeded acceptable limits for Pb, Ni, and Cd [49].

Human activities such as traffic, industry and modern agriculture are also the reasons for honey contamination by toxic metals (particularly Ni and Cr). This situation occurs when nectar is collected by honeybees in the flora of serpentine soils with elevated Ni content [50]. The production of paddy rice at serpentine sites in Taiwan has been revealed as the main source of Ni(II) exposure for humans living there [51]. Nickel can also be found in cocoa and chocolate. It is worth mentioning that Ni(II) ions are important for some plant and bacterial enzymes, however, nickel pollution is increasing in the environment [52]. In Europe, the highest levels of nickel in vegetables were found in Italy, nonetheless, the values found were of no concern for human or animal health [53]. A study by Zeinali et al. on the dietary intake of Cd, Cr, Pb, Cu, and Ni also showed that nickel content in meat was acceptable, and the highest hazard was estimated for Pb and Cd [54].

Nuapia and colleagues examined over 100 samples of meat, vegetables, and fish from African’s open markets, and showed using ICP-OES and ICP-MS methods that the content of Cd, Cr, Hg, Pb, Zn, and As exceeded WHO standards, and had a specifically negative impact on residents’ health [55].

Mounicou et al. determined that approximately 10% of lead and 10–50% of cadmium from the amount found in cocoa powder was bioavailable, and the concentration of these heavy metals depended on geographical origin [56]. Additionally, the content of heavy metals in a specific product may depend on the production stage within a plant. In the case of maize processing, germs possess the highest concentration of heavy metals [57]. Proper food preparation and processing are very important in reducing heavy-metal contamination of food products. Many beverages are polluted by heavy metals including lead, cadmium, and arsenic [58]. Even juicing vegetables and fruits, brewing coffee or extracting ginseng can change the heavy-metal concentration in the product [59].

The best way to limit heavy metal pollution is to decrease their influx into the environment and to control their concentration from the cultivation process to the consumption stage. Increased awareness and expansion of knowledge regarding the impact of heavy metals on health and possible paths to reduce and eliminate such pollutants should be the focus of future research.

## 3. Heavy Metals in Cosmetics

Heavy metals, which are present particularly in colored cosmetics, may be there either intentionally, or as impurities introduced through raw materials that are used in the production process. Such contamination may stem from improper treatment of the raw material or the production process [60,61].

According to Regulation No 1223/2009 of the European Parliament and of the Council, the presence of heavy metals (comprising Cd, Pb, As, Ni, Cr and Hg) in cosmetic products is considered dangerous for human health [62]. However, some of these elements are added to cosmetics in order to support the proper metabolic and physiological functioning of the skin, and the regulations clearly state the permissible content of these heavy metals. Among the products that contain the above-mentioned metals are those that are applied to the mucous membrane, such as lipsticks and lip glosses, beauty creams and lotions, but also hair care preparations [63,64,65].

The presence of lead in cosmetics is strictly prohibited in cosmetics in accordance with the provisions of the above-mentioned act [66]. It can be introduced into the body through the digestive and respiratory tracts or absorbed through the skin or mucous membranes. Research has shown that permanent exposure to even low levels of lead can cause eczema and skin contact allergies.

The use of eye creams, as well as color cosmetics that contain lead, may contribute to high lead concentrations in the blood [67,68]. Łodyga-Chruścińska et al. showed that two of the twelve tested cosmetics had lead concentrations that exceeded the limit proposed by FDA (maximum level of 20 ppm) [60]. In the Middle East, Africa, and Asia, a traditional cosmetic (kohl) based on antimony or galena stone (PbS_2_) is still popular. The popularity is amplified by the belief that it has a therapeutic effect on the eyes and for this reason it has been used also to treat children and infants [69]. Some of the 12 kohl-based cosmetics from German and Spanish markets analyzed recently by Navarro-Tapia et al. had excessive levels of lead, cadmium and arsenic [69].

Arsenic is used as a pigment in color cosmetics (eyeshadows, lipsticks) and is also present in many cosmetic and skin care products, such as lotions. Unlike other cosmetic ingredients, color additives must have FDA approval for their intended uses in USA. For arsenic the limit is 3 ppm [70]. The use of arsenic in its inorganic form may have many side effects, such as fatigue, nausea, vomiting, skin diseases and cancer [71,72]. In a study conducted in 2017 on 20 different types of cosmetics, arsenic was detected in skin foundation, lip balm, skin whitening cream, and hair dyes. The highest concentration of arsenic was found in lip balm (19.55 ppm) [73,74]. Skin foundations and lightening creams are also sources of another toxic metal—mercury.

Mercury may be found in cosmetics in two forms: organic (thimerosal), used as a preservative, and inorganic (HgCl_2_), used in skin lightening creams, which can be combined with other elements, such as oxygen, sulfur and chlorine [75]. However, mercury compounds are allowed in cosmetics only as preservatives. Numerous reports have indicated that mercury is still used in cosmetic products in countries such as Mexico, US, Africa and Asia [76,77,78]. Many of the tested cosmetics exceeded the acceptable limit established by the WHO and the FDA (it should be no more than 65 parts per million (ppm) in the finished product) [71]. Scientific studies have shown that people exposed to skin-lightening creams exhibit increased levels of mercury in their body [79].

Therefore, long-term use of cosmetics that contain even small amounts of mercury can cause kidney damage, skin damage in the form of discoloration or allergic changes, as well as peripheral neuropathy [80]. In 2021, Lara-Torres et al. showed that the tested cosmetics did not exceed the limits suggested by FDA, but completely violated the Regulation of the European Parliament [81]. Reports of mercury poisoning have also been documented in pregnant women and in children. A case has been described of a four-year-old girl from Iraq, who after three months of applying whitening cream, showed symptoms of mercury poisoning. The child’s symptoms included loss of appetite, seizures, weight loss, weakness, and body rash. Her urine mercury levels were significantly above the generally accepted standard. Additionally, studies conducted on women in the third trimester of pregnancy who used whitening creams displayed mercury concentrations of 15.16 µg L^−1^ in their blood [82,83]. Skin-lightening products are extremely popular among the Indian and African population, as well as US, where, inter alia, Nephrotic Syndrome has been reported [84,85].

Cadmium can be present in inorganic pigments found in cosmetic products. It has been shown that cadmium can concentrate in bones, kidneys and teeth. Studies conducted on rats indicate that contact with cadmium results in fetal growth inhibition and teratogenicity [86,87,88]. Lara-Torres et al. showed that cadmium is present in both Chinese and European cosmetics. Cadmium concentration in lipsticks was at the level of 0.07 mg kg^−1^ in samples from China and 0.01 mg kg^−1^ in samples from Europe [77]. The FDA has not yet defined the maximum cadmium concentration in cosmetics. However, Regulation (EC) No 1223/2009 prohibits the use of cadmium and its compounds in the cosmetics [67]. The IARC (International Agency of Research on Cancer) classified cadmium and lead as group 2A of carcinogenic substances. Cosmetic products, especially lipsticks, can be dangerous to health, as average women inadvertently swallow as many as 4 pounds of lipstick during their lifetime [89]. Cadmium can also be found in rinse-off products. Long-term use of these products may lead to skin rashes, epithelium problems, and distortion of other organs [90].

Skin dermatitis can be caused also by nickel present in jewelry and cosmetics. Nickel usage in cosmetic products is prohibited by European Parliament [67], however, the available data show that it can be found in many types of color cosmetics produced and used in various parts of the world [62]. Studies show that it is detected mainly in lipsticks and powders [60,91].

Generally, toxic metals present in cosmetics may act directly on the skin or indirectly by absorption through the skin into the blood, leading to bioaccumulation and toxic effects in various organs. Below we will review their impact on the human body on the molecular level.

## 4. The Impact of Toxic Metals on Human Enzymatic Pathways

The competition between metal ions for protein-binding sites can cause problems in the transport of some molecules and catalysis of chemical reactions in human organisms. The composition of the immediate environment of the metal-binding site is related to metal selectivity. However, the selectivity is different for each protein, so it should be studied at the single-protein level [92,93,94]. The important aspect of metal ions’ impacts on the human body are metal-mediated protein–protein interactions. Understanding these interactions is central to understanding the molecular details of heavy-metal impact on human health [95].

Mechanisms to maintain metal homeostasis in the cells involve mainly cysteine (Cys-)-rich metal-binding peptides such as glutathione (GSH) and proteins such as metallothioneins (MTs), as well as phytochelatins (PCs) in plants and microorganisms [96]. Metallothioneins, discovered in 1957 as cadmium-binding proteins, can bind and sequester heavy metals due to their high cysteine content [97]. They also exhibit high potential for other heavy metals, which makes them good candidates for protein-based metal biosensors [98]. In addition, other proteins and several lysosome-like organelles participate in homeostasis, acting as storage sites and buffering cytosolic metal levels in eukaryotes [99]. Metallothioneins seems to be protective proteins produced in response to different kinds of stresses.

Cadmium and lead, due to their biophysicochemical similarities to calcium, magnesium and other divalent minerals, have the ability to mimic essential metals and/or to replace them at their specific sites. These ions bind to magnesium, zinc and calcium specific sites in calmodulin [100], protein kinase C [101], troponic C [102] and synaptic proteins [1]. However, Cd(II) and Pb(II) are unable to mediate some vital functions, which makes them fatal to mammalian cells. They belong to so called “soft metals” and form the most stable complexes with the mixed N-S donor atom ligand. Cd(II) complexes would be more stable than those of Mg(II) and Ca(II) for complexes involving O-O donor atom chelation (oxalate) [103]. Divalent cadmium and calcium ions have similar physicochemical properties in aqueous solutions. These ions are both able to exert strong electrostatic forces on biological macromolecules because of the similar charge/radius ratios (Ca(II) = 2.02 e/Å, Cd(II) = 2.06 e/Å) [103].

This also means that cadmium is less readily hydrated than magnesium cations, which resemble calcium behavior. For this reason, cadmium cations might be taken up by voltage- or receptor-operated Ca(II) channels [103]. However, it appears that Cd(II) may also be transported by SOCs in electrically non-excitable cells.

Recent investigations on mouse renal tubular epithelial cells have shown that cadmium can induce Ca(II) release from *endoplasmic reticulum* (ER) stores through the phospholipase C (PLC)-IP_3_ pathway, leading to ROS generation [104]. It has been deciphered that cadmium ions cause kidney damage by increasing intracellular Ca(II) levels, mitogen activated protein kinase (MAPK) activation, and by altering the activities of ion transporters in renal cells [105]. The elevation of intracellular Ca(II) associated with ROS generation is also responsible for Cd-induced apoptosis and cytotoxicity in immune cells [106]. It has been suggested that activation of CaSR by calcimimetics can prevent Cd-induced apoptosis by restoring proper Ca(II) levels, switching the MAPK pathways and reducing ROS generation [105]. On the other hand, cadmium ions can deplete calcium stores over longer periods of time [103]. Cadmium has been described as a metalloestrogen due to its ability to bind to estrogen receptor alpha, and to activate this receptor in the absence of estrogen, leading to breast cancer development [107]. Cadmium has also an adverse impact on the nervous system. Cd(II) neurotoxicity has been shown to be connected with the cleavage of caspase-9 and caspase-3 proteases, and decreased mitochondrial membrane potential [103]. Toxic effects on calcium homeostasis could be caused by a decrease in Ca(II) efflux. Visser and coworkers have shown that cadmium ions inhibit Ca-ATPase activity in the nanomolar range [108].

Lead remains one of the most studied toxic elements due to the extensive exposure of humans to lead since ancient times, and because of Pb(II)’s toxic, especially neurotoxic, effects [7]. Lead was shown to block voltage-activated and receptor-operated calcium channels in invertebrate neurons as well as in mammalian neurons. In mammals, lead ions were shown to block N-, L- and T-type voltage-activated calcium channels [109]. Pb(II) exposure impairs synapse development, which may have lasting consequences on downstream signaling [110].

Interestingly, Pb(II) levels in bone (chronic exposure) rather than blood (short exposure) are good biomarkers for lead toxicity on the development of the nervous system [111]. Pb-induced modifications in either oxidative stress or homocysteine levels can cause disorders in epigenetic processes [111]. Aimo and Oteiza have shown that zinc deficiency accelerates the action of Pb(II), promoting oxidative stress, MAPK and AP-1 (activator protein-1) activation, and decreased neuronal cell viability [112].

Lead ions can cause deterioration of mitochondrial morphology and mitochondrial function, resulting in cell apoptosis. The mitochondrial permeability transition pore (mPTP) is induced mainly through ROS production [113].

As mentioned above, high levels of cellular calcium can also influence mPTP occurrence. However, in normal conditions, the cellular and mitochondrial levels of ROS are safe, and participate to the vital activity of the cell. The production of ROS becomes detrimental for the cell under acute and chronic cellular stress conditions [114]. Nonetheless, lead has been shown to cause ROS accumulation that can provoke energy metabolism impairment and DNA alteration, including fragmentation, rearrangements, deletions, and point mutations [113]. Nondividing cells such as neurons can be carriers of long-term epigenetic changes induced during their development. A recent study, in consistency with previous data, reveals an association between early-life exposure to lead and DNA hypomethylation [115]. Bijoor and coauthors revealed that even low blood levels of lead can cause alterations in neurotransmitters, such a norepinephrine (NE) and its metabolite [116].

Disruptive effects of lead ions on mitochondrial respiratory complexes has been proposed also as the cause of Pb(II)-induced liver toxicity. The mechanism of that toxicity employs oxidative stress, which leads to mitochondrial dysfunctions and even cell death signaling via PTP opening and cytochrome c release [117].

The chemical forms of mercury include metallic (Hg°), mercuric (Hg(II)) and organic compounds. Metallic mercury is slightly absorbed from the intestine, however its vapor is readily absorbed from the lungs and it can pass the blood–brain barrier (BBB), and placenta [15]. In the body it is oxidized to Hg(II). Divalent mercury has also been shown, similarly to above-mentioned metals, to have the ability to induce oxidative stress leading to disturbances in calcium homeostasis [118]. In some countries, HgCl_2_ is still added to lightening creams as the active ingredient. It inhibits tyrosinase (an enzyme controlling the production of melanin) activity irreversibly by replacing the copper cofactor [119].

Organic mercury compounds (comprising dimethylmercury, ethylmercury, and phenylmercury) are more toxic than inorganic forms [120]. Methylmercury has a high affinity for sulfhydryl (thiol, -SH) moieties. The binding of methylmercury to Cys can mediate multiple toxic effects of this metal, especially inhibitory effects on enzymes. In blood plasma, albumin is the main Hg-binding protein.

In addition to albumin, mercury binds to multiple Cys-containing enzymes (e.g., including manganese-superoxide dismutase (Mn-SOD), arginase I, and sorbitol dehydrogenase) involved in multiple processes [121]. However, the existing knowledge on the role of mercury-SH binding in Hg toxicity is scarce and insufficient. In the brain, binding of mercury to selenium or SH-groups may contribute to remaining brain deposits for a long time, as long as 20 years [26]. According to the WHO, ethylmercury (thiomersal used as a preservative) is broken down by the body quickly and does not accumulate, however, there are some reports showing that ethylmercury can also cross the BBB [122]. Moreover, simultaneous exposure to both MeHg and EtHg can have synergistic effects on developing and mature humans [123]. EtHg-induced toxicity represents an issue which needs to be solved by more studies and their results.

Arsenic’s damaging action depends on the bioavailability, chemical form (metalloid, inorganic or organic), and metabolism of the human body. Oxidative stress has a major role in arsenic-induced toxicity [124]. The most toxic oxidative states of As are the inorganic arsenite(III) and arsenate(V) states. Arsenite is considered to be a more toxic inorganic form compared to arsenate, and the most toxic form of arsenic is Arsine gas (AsH_3_). Inhalation of over 10 ppm can be lethal [125]. Chronic exposure to elevated concentrations of arsenic has been associated with the prevalence of several cancers and increased risk of a number of noncancerous effects. Arsenic binding to a specific protein can change the conformation and function of the protein, and thus can alter its interaction with other functional proteins [126]. It is suggested that the main source of carcinogenicity and toxicity of arsenic is due to its reduction to arsenite in the body.

Arsenite was shown to react with thiol-containing molecules, e.g., glutathione, which leads to the formation of the terminal metabolite, dimethylarsinic acid (DMA), which is excreted [127]. On the other hand, some reports show that metabolically generated methylated species of arsenic can be even more toxic than their parent species. It has been suggested that rather than detoxification, methylation appears to function as activation pathways through which enhancement of As toxicities is achieved. Toxicities of metabolically generated methylated species vary with respect to their extent of undergoing methylation [128].

In animal models, As neurotoxicity was attributed to the disturbances in the expression of N-methyl-d-aspartame (NMDA) receptor and alterations in molecular expression of postsynaptic signaling proteins [129]. Arsenic can also substitute phosphate in the anion exchange transport system and replace the phosphate in the enzymatic reaction, which diminishes the ATP formation in vitro [130].

As mentioned above, there are also heavy metals which are needed in small amounts for the proper functioning of human enzymes (e.g., Cu, Mn, Zn as trace elements) or are needed for other organisms but not vertebrates (e.g., Ni).

Although nickel is an essential component for some bacteria, fungi, invertebrates and plants, in humans it can cause allergies, bronchitis, and nose and lung cancers (as was reported in workers in nickel refinery) [131]. Interestingly, nickel can be also indirectly responsible for stomach ulcers and cancers, being the important cofactor in *H.pylori’s* bacteria enzymes: [NiFe]-hydrogenase and urease [132]. In these enzymes, nickel is coordinated to His, Cys, and Asp residues [132,133]. Nickel has a high affinity towards proteins and peptides containing thiol groups [132]. After nickel coordination by certain endogenous ligands, the nickel ions can provoke formation of reactive oxygen intermediates, causing the redox imbalance which may be related to oncogenic stimulation [2]. Moreover, the repair and the replication of DNA can be inhibited by the presence of Ni(II) [2]. However, the most common health impact of nickel in humans is contact allergies. They are associated with nickel’s ability to bind to amino acid residues and to form Ni-complexed proteins [134]. Recently, a study on the response of adult human epidermal keratinocytes to nickel nanoparticles revealed that exposure to them leads to the release and accumulation of Ni(II) ions in the cytosol and the biosynthesis of a nickel-binding molecule related to the p63-regulated gene 1 protein [135]. Usually, the proteins comprising histidine-rich motifs can serve as Ni-accessory proteins to bind and sequester Ni(II) ions inside cells, depending on the surrounding conditions [132,136].

## 5. Methods of Bioremediation

According to the WHO/EUROPE, the tolerable weekly intake values for Hg, Pb, Cd, and As metals are 0.0016, 0.025, 0.007, and 0.015 mg/kg body weight, respectively [137]. Many efforts are made to minimize the threat of heavy metal environmental pollution to human health. However, there are still places where elevated levels of heavy metals, above the WHO limits for agricultural soils and wastewater, are present [138,139]. Recent studies have focused on the bioremediation of heavy metals instead of the chemical processes of detoxification [140,141].

Bioremediation is economically achievable and more beneficial for the environment.

**Figure 2:**
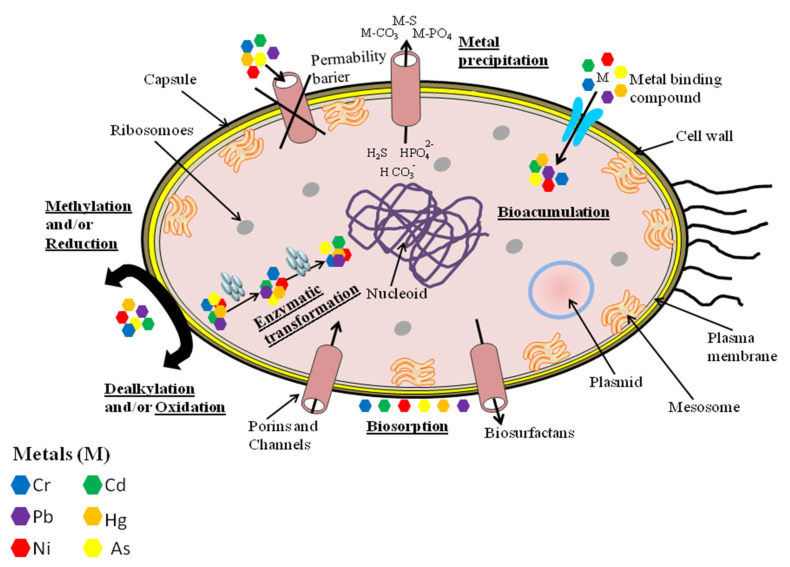
General mechanisms utilized by bacteria, and plant cells for metal resistance and detoxification, which can be adapted in bioremediation techniques. Based on [130].

Some metal ions, which do not have physiological functions in humans, can be important for bacteria. One of them is nickel—the cofactor of urease and Ni-Fe hydrogenase enzymes [132]. Nonetheless, most metals showing toxicity to animals also are hazardous to microorganisms. Even Ni(II) is toxic to bacteria at elevated concentrations. Bacteria have developed certain strategies to regulate metal homeostasis within the cell (Figure 2).

These strategies include: (1) metal exclusion and active transport away from the cell, (2) enzymatic detoxification to less-toxic forms, (3) intra- or extracellular sequestration, to name a few [142]. Many microorganisms have stress-resistance genes in the plasmid and chromosomal systems. They help to reduce the sensitivity of cellular targets to toxic metal ions. Bacteria and fungi do not directly degrade heavy metals but convert the valence states of these pollutants into less hazardous or immobile forms. This ability is used in bioremediation techniques. In situ bioremediation uses naturally occurring bacteria to degrade contaminants in soil or water. In ex situ bioremediation, polluted soil is located in a lined above-ground treatment area of an indigenous microbial community [143,144].

In a plant-based approach (phytoremediation) the metal-binding proteins are used to remove heavy metals from the environment or to reduce their toxic effect [128]. Important roles have been played in this process by peptides and small proteins, such as phytochelatins and metallothioneins. It is well known that toxic metals inhibit plant growth, which can lead to their early mortality. The toxicity of these metals and other pollutants can be reduced by endophytic microbes, such as *Pantoea* sp., *Pseudomonas* sp., or *Flavobacterium* sp. Recent studies have also revealed that *Cyanobacteria* are efficient in the removal of heavy metals from various waste yards and soils [145].

These are only a few methods utilized to remediate heavy metal contaminated soil, water, and sediments. A much wider description of this topic can be found in a recent review of Dhaliwal et al. [146].

## 6. Conclusions

This work presents a review of the mechanisms of action of the most dangerous heavy metals (Hg, Pb, Cd, As, and Ni). Combined exposure to toxic metals and other xenobiotic substances can boost the toxic hazard for human health.

Toxic metals pose a threat to the human body due to a few characteristics: (1) These metallic elements are considered systemic toxicants—they can cause adverse health effects in organs distant from the entry point and render there for a long time; (2) They can replace essential metals from their binding sites in proteins and enzymes. Moreover, one disorder in metal homeostasis often affects the distribution of another metal and may lead to disturbances in enzymatic pathways; (3) Some of these elements (cadmium, mercury, lead, and arsenic), even in tiny amounts, have deleterious effects in humans, causing acute and chronic toxicities; (4) In recent decades, human exposure to these elements has risen dramatically as a result of their use in many domestic, industrial, technological, and agricultural applications; (5) Their toxicity depends on the form (organic or inorganic) and the manner of exposure; (6) Toxic metals can also interact with nuclear proteins and with DNA, causing site-specific damage and the production of reactive oxygen species.

Metals are nonbiodegradable. Organisms may deposit them in an insoluble form to be excreted from the organism or detoxify them by binding them to “accessory” proteins. The importance of small proteins, such as metallothioneins, calprotectin and phytochelatins in detoxification processes has to be stressed.

Nowadays, there are a lot of methods to remove heavy metals from the soil, water, and air. Many researchers are developing further chemical methods of detoxification such as bioremediation and phytoremediation. Simple and cheap methods are of great importance, especially for developing countries. Increased awareness and expansion of knowledge regarding the impact of heavy metals on human health and possible ways to reduce and eliminate such pollutants should be the focus of future research.
